# Effects of pretransplant peritoneal vs hemodialysis modality on outcome of first kidney transplantation from donors after cardiac death

**DOI:** 10.1186/s12882-018-1013-3

**Published:** 2018-09-17

**Authors:** Xiajing Che, Xiaoqian Yang, Jiayi Yan, Yanhong Yuan, Qing Ma, Liang Ying, Minfang Zhang, Qin Wang, Ming Zhang, Zhaohui Ni, Shan Mou

**Affiliations:** 1 Department of Nephrology, Molecular Cell Laboratory for Kidney Disease, Renji Hospital, School of Medicine, Shanghai Jiao Tong University, 160 Pujian Road, Shanghai, 200127 China; 2 Transplantation Center of Ren Ji Hospital, School of Medicine, Shanghai Jiao Tong University, 160 Pujian Road, Shanghai, 200127 China

**Keywords:** Dialysis modality, Donor after cardiac death (DCD), Hemodialysis (HD), Outcomes of kidney transplantation, Peritoneal dialysis (PD)

## Abstract

**Background:**

The effect of pretransplant peritoneal dialysis (PD) or hemodialysis (HD) modality on outcomes of kidney transplantation (KT) for end-stage renal disease (ESRD) is debatable. We evaluated the outcomes these modalities in KT from donor after cardiac death (DCD).

**Methods:**

A cohort of 251 patients on HD, PD or pre-emptive who underwent first KT from DCD between January 2014 and December 2016 were prospectively analyzed to compare for outcomes on recovery of renal function, complications as well as patient and graft survival. The patients were followed till August 2017. Data on 104 HD and 98 PD were available for final comparative outcome analysis, 5 pre-emptive were analyzed as the control group.

**Results:**

Both HD and PD group patients were well matched for demographic and baseline characteristics. The follow-up period was 12.5 (3.0, 22.0) months in HD and 12.0 (6.0, 20.0) months in PD patients. Post-transplant renal functions between the two groups showed no differences. Among PD patients, 16 (16.3%) suffered delayed graft function, versus 19 (18.3%) in HD, with no statistical differences (*p* = 0.715). Complications of acute rejection, infections were comparable between the groups. The patient survival, graft survival and death-censored graft survival were similar for HD and PD after adjusting for other multiple risk factors.

**Conclusions:**

Our results indicate that outcome of first KT from DCD is not affected by pretransplant dialysis modality of PD or HD in aspects of recovery of renal function, complications as well as patient and graft survival.

## Background

Peritoneal dialysis (PD), hemodialysis (HD) and kidney transplantation (KT) are three main renal replacement therapies for end-stage renal disease (ESRD) and KT with advances in technology and immunosuppressants is preferred for the recovery of renal function and the improvement of life quality [1, 2]. The availability of donor kidney has restricted the transplant and dialysis is essential while waiting for KT.

While awaiting KT, 30–40% of patients can be effectively treated by PD which is far away from the actual 11% and many suitable PD candidates are treated with HD [3]. Controversies on pretransplant dialysis modality continues with reported increased risks of early graft failure in PD patients [4]. Recent studies show equivalent outcomes for PD and HD [5–7]. Yet, other studies indicate better outcome of PD for patient survival, graft function as well as the delayed graft function (DGF) [8–13].

More studies are needed to clarify the identical or even better function of PD compared with HD. Therefore, we conducted this prospective cohort study to compare the effects of pretransplant HD vs PD on outcomes of renal function, post-transplant complications, graft as well as patient survival of first KT from Donor after cardiac death (DCD).

## Methods

### Study population

This was a prospective cohort study of ESRD (defined as eGFR< 15 ml/min/1.73 m^2^) patients who received their first kidney transplantation from DCD between January 2014 and December 2016 in renji hospital affiliated to School of Medicine of Shanghai Jiaotong University, a hospital at Pudong New District, Shanghai, China. During the transplantation, the technical issues that may affect the outcome of transplant like organ transplantation, preservation as well as surgical operation were all performed by same transplant team in our hospital. DCD was defined as awaiting cardiac arrest after withdrawal of life-supporting treatment in the intensive care unit.

**Fig. 1:**
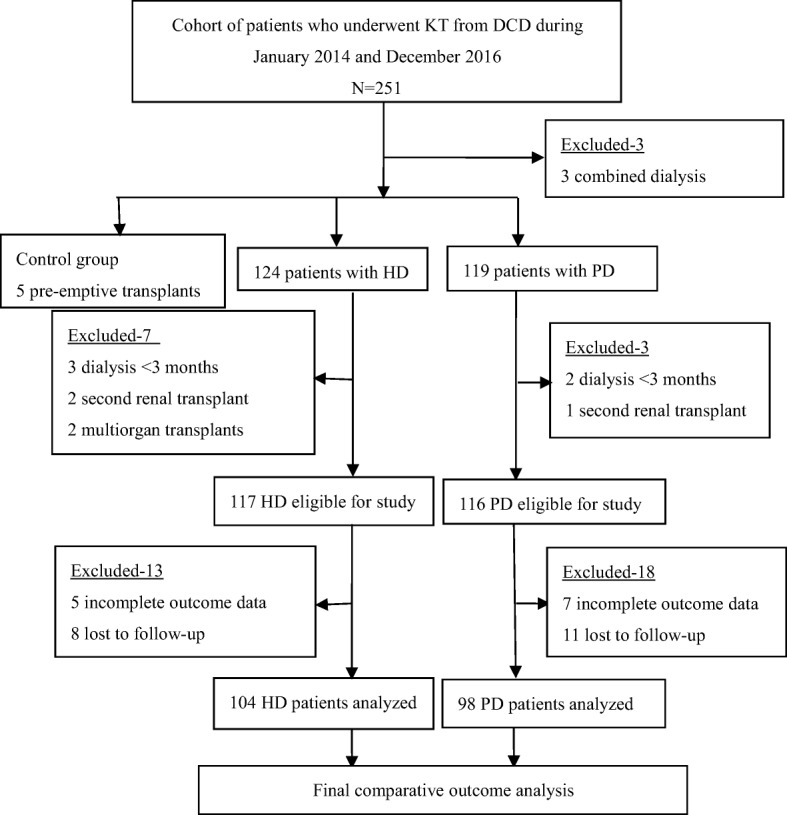
Flowchart of patients who received KT from DCD. *ERSD* end stage renal disease, *HD* hemodialysis, *PD* peritoneal dialysis, *KT* kidney transplantation, *DCD* donor after cardiac death

Patients above 18 years of age who had been on the same dialysis modality (hemodialysis or peritoneal dialysis) for at least 3 months without a switch or underwent transplantation before the initiation of dialysis (pre-emptive kidney transplantation, PKT) were included. Patients who were living donor transplant, second KT or multiple organ transplants and lost to follow-up were excluded. Follow-up was terminated on August 2017. Finally data on 104 HD and 98 PD group met the inclusion criteria and were included for comparative outcome analysis. 5 PKT patients were analyzed as the control group (Fig. 1).

The protocol of this study was reviewed and approved by the Ethics Committee of Renji hospital, and patients were included only after signing informed consent.

### Data source

Donor variables included age, gender, BMI, blood group, percentage of hypertension, mean time of intensive care unit(ICU) stay, HLA mismatching, estimated glomerular filtration rate (eGFR) and the causes of death. The baseline variables of recipients in two groups of patients on hemodialysis or peritoneal dialysis included age, gender, body mass index (BMI), blood group, duration time on pretransplant dialysis, post-transplant hospital stay and follow-up time, preoperative medical condition, percentage of anti-hypertensive drugs required, percentage of hepatitis B virus (HBV) infection, native kidney diseases, pretransplant urinary volume, percentage of anuric patients and immunosuppresion therapy. The laboratory parameters of white blood cell count, creatinine, cholesterol, triglyceride of both donors and recipients were collected. The percentage of neutrophilic granulocyte and lymphocyte, haemoglobin, serum urea nitrogen, serum uric acid, serum albumin, alanine aminotransferase (ALT), blood glucose, serum potassium, serum sodium, parathyroid hormone, serum calcium, serum phosphate, low-density lipoprotein (LDL), high-density lipoprotein (HDL), and lymphocyte subtypes were also obtained from recipients. Among which the eGFR was estimated using the chronic kidney disease epidemiology collaboration (CKD-EPI) equation which was calculated according to the gender, the serum creatinine, the age and the race.

Post-transplant variables included renal function (serum creatinine, 24 h urine volume and the eGFR), haemoglobin, serum albumin, cholesterol, triglyceride, serum calcium and serum phosphate. The postoperative complications during hospitalization of delayed graft function (DGF), acute rejection (AR) and surgical complications including the urinaryfistula, hydronephrosis and hematoma after tranplantation were recorded. The infective complications including viral infection(cytomegalovirus, JC virus, BK virus, varicella zoster virus), fungal infection as well as bacterial infection(tuberculosis, urinary tract infection, acute bacterial pneumonia, gastrointestinal infection) were recorded during hospitalization and the whole follow-up time. The DGF was defined as the requirement for dialysis in the first week after transplantation, or serum creatinine level increased, remained unchanged, or decreased by less than 10% per day immediately after surgery [14, 15]. All patients with biopsy-proven acute rejection and those with features of antibody-mediated rejection, with borderline changes and allograft dysfunction who received treatment for acute rejection were considered to have rejection [14, 15]. The patient, graft and death-censored graft survival were compared between HD and PD groups. The causes of patient mortality and graft failure were recorded. Death-censored graft failure was defined as suffering graft failure without death.

### Statistical methods

The statistical analysis was performed by using the SPSS 22 version software. All numeric variables were tested for normality of their distribution. Independent samples t-test and Mann-Whitney U test were respectively used for analyzing data whose distribution are normal and abnormal. Results are described as mean ± standard deviations (SD) for normally distributed data, median and interquartile range (IQR, pp. 25–75) for abnormally distributed data. The Chi square or Fisher’s exact tests was utilized to compare the categorical variables between the two groups. The results were expressed in numbers and relative frequencies [n(%)].

The patient and graft survival were calculated from the date of transplantation to the endpoints of the study. The univariate and multivariate analysis were conducted for risk factors for graft failure in HD and PD groups. The univariate analysis was conducted to study the risk factors of patient mortality and graft failure. Variables whose *p* < 0.05 in the univariate analysis or clinical meaningful were enrolled into multivariate analysis. The cox proportional regression models were used to assess the relative risks.

Variables of *p* values < 0.05 were considered to be statistically significant. All statistical tests were two-tailed.

## Results

### Baseline clinical characteristics

The baseline information of recipients and donors in HD, PD and PKT group was analyzed. Among them, PKT group was analyzed as the control group. Patients in both PD and HD groups were comparable for demographic and clinical characteristics (Table 1). The pretransplant laboratory parameters in PD group were lower compared with HD group with regard to haemoglobin, serum albumin, serum potassium and serum calcium (*p* < 0.05). On the contrary, the serum creatinine, serum urea nitrogen and serum uric acid were higher in PD group (*p* < 0.05) (Table 2). Donors characteristics was comparable for recipient in both PD and HD groups (Table 3).

**Table 1:** Demography and Clinical Characteristics of ESRD patients on pretransplant pre-emptive, HD or PD who received KT from DCD

Characteristics	PKT group (*n* = 5)	HD group (*n* = 104)	PD group (*n* = 98)	*p*-value
Age(years)	36.2 ± 10.1	42.4 ± 9.7	39.5 ± 11.6	0.053
Male, n (%)	4(80.0)	65(62.5)	56(57.1)	0.437
BMI (kg/m^2^)	22.1(20.2,26.1)	21.2(18.9,23.6)	21.5(19.9,23.8)	0.470
Blood group (A:B:AB:O)	2:2:0:1	27:26:13:38	27:29:8:34	0.706
Duration on dialysis (months)	0(0,0)	15.5(6.0,36.0)	24.0(6.0,36.0)	0.583
Hospital stay (days)	17.0(12.5,19.5)	20.0(15.0,26.0)	18.5(15.0,29.0)	0.337
Follow-up time (months)	19.0(10.5,35.0)	12.5(3.0,22.0)	12.0(6.0,20.0)	0.961
Preoperative medical condition, n (%)				
Diabetes mellitus	0(0)	13(12.5)	6(6.1)	0.121
Cardiovascular disease	0(0)	5(4.8)	2(2.0)	0.490
Hypertension	5(100)	91(87.5)	90(91.8)	0.313
Antihypertensive agents, n (%)	5(100)	76(73.1)	75(76.5)	0.572
HBV (+)	0(0)	9(8.7)	8(8.2)	0.900
Cause of end-stage renal disease, n (%)				0.188
Glomerulonephritis	4(80)	68(64.8)	64(66.0)	
Diabetes	0(0)	5(4.8)	1(1.0)	
Hypertensive nephrosclerosis	0(0)	2(1.9)	1(1.0)	
Polycystic kidney disease	1(20)	6(5.7)	1(1.0)	
Chronic pyelonephritis	0(0)	1(1.0)	2(2.1)	
Others	0(0)	2(1.9)	6(6.2)	
Unknown	0(0)	21(20.0)	22(22.7)	
Pretransplant urinary volume (ml/24 h)	2000(1750,2000)	200(100,500)	500(100,1000)	0.073
Anuric patients (%)	0(0)	19(18.3)	18(18.4)	0.986
Immunosuppresion therapy, n (%)				0.310
St + FK + MMF	5(100)	97(93.3)	95(96.9)	
St + CyA	0(0)	2(1.9)	0(0)	
St + CyA + MMF	0(0)	5(4.8)	3(3.1)	

*P*-value, between hemodialysis and peritoneal dialysis group

*ERSD* end stage renal disease, *HD* hemodialysis, *PD* peritoneal dialysis, *PKT* pre-emptive kidney transplantation, *KT* kidney transplantation, *DCD* donor after cardiac death, *BMI* body mass index, *HBV* hepatitis B virus, *St* steroids, *FK* tacrolimus, *MMF* mofetil mycofenolate, *CyA* cyclosporine A

**Table 2:** Pretransplant Laboratory Parameters of Kidney Recipients

Laboratory Parameters	PKT group (*n* = 5)	HD group (*n* = 104)	PD group (*n* = 98)	*p*-value
Serum white blood cell (10^9^/L)	6.2 ± 1.4	7.1 ± 1.8	7.1 ± 2.0	0.832
Neut %	62.6 ± 8.2	68.1 ± 7.4	68.8 ± 8.6	0.539
Lymph%	21.6(19.6,31.0)	21.6(17.4,26.2)	20.5(15.8,26.0)	0.168
Haemoglobin (g/L)	101.4 ± 8.9	115.8 ± 19.8	105.1 ± 18.7	< 0.001*
Serum potassium (mmol/L)	3.5(2.9,4.3)	4.3(3.9,4.8)	3.8(3.3,4.3)	< 0.001*
Serum sodium (mmol/L)	140.0(137.5142.0)	139.0(138.0,141.0)	139.0(136.0,141.0)	0.213
PTH (pg/ml)	202.4(149.7707.4)	221.0(87.8431.2)	216.3(134.1469.0)	0.198
Serum calcium (mmol/L)	2.1 ± 0.2	2.4 ± 0.2	2.3 ± 0.2	0.008*
Serum phosphate (mmol/L)	1.8(1.7,3.0)	1.7(1.3,2.3)	1.8(1.3,2.3)	0.864
Serum creatinine (umol/L)	751.0(706.5785.3)	827.2(647.0,1068.0)	1104.1(827.8,1426.7)	< 0.001*
Serum urea nitrogen (mmol/L)	36.1 ± 13.7	18.7 ± 6.6	21.9 ± 6.4	0.001*
Serum uric acid (μmol/L)	533.0(372.5642.0)	346.0(290.3424.5)	408.5(384.8462.5)	< 0.001*
Serum albumin (g/L)	48.9 ± 4.2	48.4 ± 4.4	42.7 ± 4.3	< 0.001*
ALT(U/L)	14.0(7.0,19.4)	14.0(9.7,20.0)	16.0(11.0,20.5)	0.151
Blood Glucose (mmol/L)	4.5(3.3,5.9)	4.4(3.8,4.9)	4.3(3.6,5.0)	0.983
Cholesterol (mmol/L)	4.3 ± 0.9	4.8 ± 1.1	5.1 ± 1.2	0.086
Triglyceride (mmol/L)	1.8(1.2,3.7)	1.6(1.1,2.9)	1.7(1.2,2.6)	0.683
LDL (mmol/L)	2.1(2.0,2.8)	2.5(2.2,3.1)	2.9(2.2,3.6)	0.057
HDL (mmol/L)	1.0(0.9,1.2)	1.1(0.9,1.6)	1.1(0.9,1.4)	0.675
Lymphocyte subtypes(%)				
CD3^+^(%)	76.3(70.2,84.2)	70.8(62.9, 76.6)	71.0(66.0, 75.9)	0.868
CD4^+^/CD8^+^	1.7(1.2,1.9)	1.6(1.2, 2.1)	1.6(1.2, 2.0)	0.766
CD19^+^(%)	6.8(5.5,11.0)	11.1(7.7, 13.6)	9.5(7.2, 13.8)	0.636
CD16^+^CD56^+^(%)	13.1(6.8,18.2)	14.6(9.9, 19.2)	5.6(10.1, 19.5)	0.742

*P*-value, between hemodialysis and peritoneal dialysis group; *, statistically significant

*PKT* pre-emptive kidney transplantation, *HD* hemodialysis, *PD* peritoneal dialysis, *PT* parathyroid hormone, *ALT* alanine aminotransferase, *LDL* low-density lipoprotein, *HDL* high-density lipoprotein

**Table 3:** Demography and Clinical Characteristics of Kidney Donors after Cardiac Death (DCD)

Characteristics	Donated to PKT (*n* = 5)	Donated to HD (*n* = 79)	Donated to PD (*n* = 83)	*p*-value
Age(years)	42.8 ± 17.0	40.3 ± 13.7	37.7 ± 16.5	0.451
Male, n (%)	5(100.0)	52(65.8)	62(74.7)	0.216
BMI (kg/m^2^)	21.8(20.4,26.5)	21.5(19.5,23.0)	21.2(19.8,24.0)	0.972
Blood group (A:B:AB:O)	2:2:0:1	13:20:9:37	18:23:11:31	0.649
Hypertension(%)	2(40.0)	36(41.8)	25(34.4)	0.355
ICU stay	2.0(1.5,21.0)	2.0(1.5,4.0)	2.0(1.0,4.5)	0.510
HLA mismatching				0.098
0–2	3(60.0)	23(29.1)	13(15.7)	
3–4	1(20.0)	8(10.1)	13(15.7)	
5–6	1(20.0)	48(60.8)	57(68.7)	
Serum white blood cell (10^9^/L)	12.7 ± 9.0	12.2 ± 5.7	14.6 ± 9.9	0.424
Serum creatinine (umol/L)	64.1(36.5,65.5)	67.5(48.7107.3)	74.0(48.0,95.0)	0.947
Cholesterol (mmol/L)	3.3 ± 1.1	3.2 ± 1.3	3.7 ± 1.6	0.429
Triglyceride (mmol/L)	1.2 ± 0.6	1.4 ± 0.7	1.3 ± 0.8	0.830
eGFR(mL/min/1.73m^2^)	110.0(98.0,133.0)	129.4(72.1215.2)	109.5(72.8209.0)	0.930
Cause of death,n (%)				0.179
Cerebrovascular accident	2(40.0)	27(34.6)	28(37.3)	
Trauma	3(60.0)	23(29.5)	26(34.7)	
Cerebral tumor	0(0)	3(3.8)	2(2.7)	
Others	0(0)	3(3.8)	8(10.7)	
Unknown	0(0)	22(28.2)	11(14.7)	

*P*-value, between hemodialysis and peritoneal dialysis group

*PKT* pre-emptive kidney transplantation, *HD* hemodialysis, *PD* peritoneal dialysis, *ICU* intensive care unit, *eGFR* estimated glomerular filtration rate

### Post-transplant renal function outcomes

**Fig. 2:**
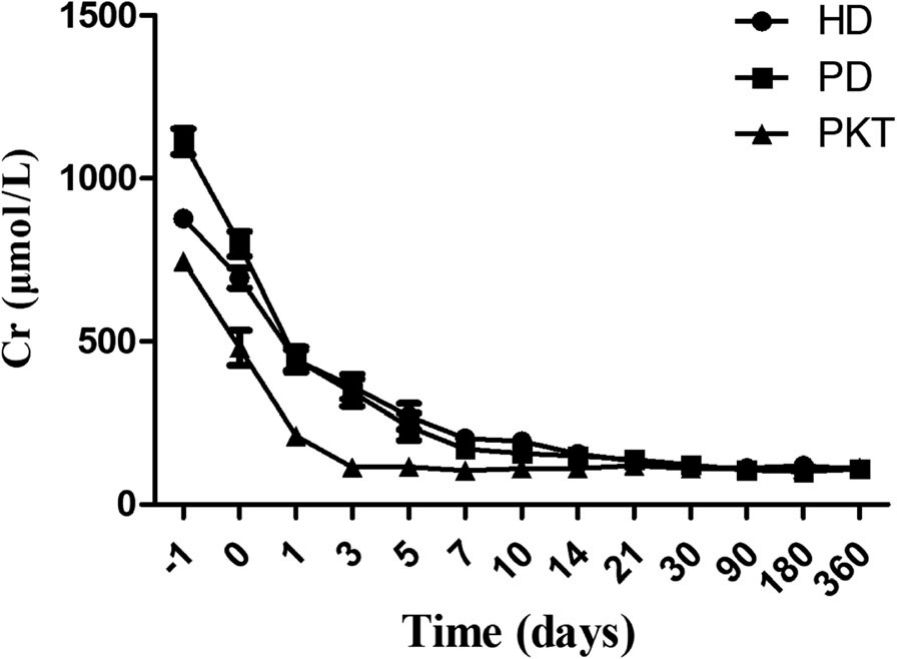
The serum creatinine from pretransplant to 1 year after transplantation in PD, HD and PKT. The horizontal ordinate refers to the pretransplant time (− 1) as well as post transplantation follow-up time. The baseline serum creatinine level is higher in PD patients compared with HD patients (*p* < 0.05). During the follow-up time, the serum creatinine level had no differences between the HD and PD groups (*p* > 0.05). PKT group was used as the control group. Data were expressed as means±S.E

**Fig. 3:**
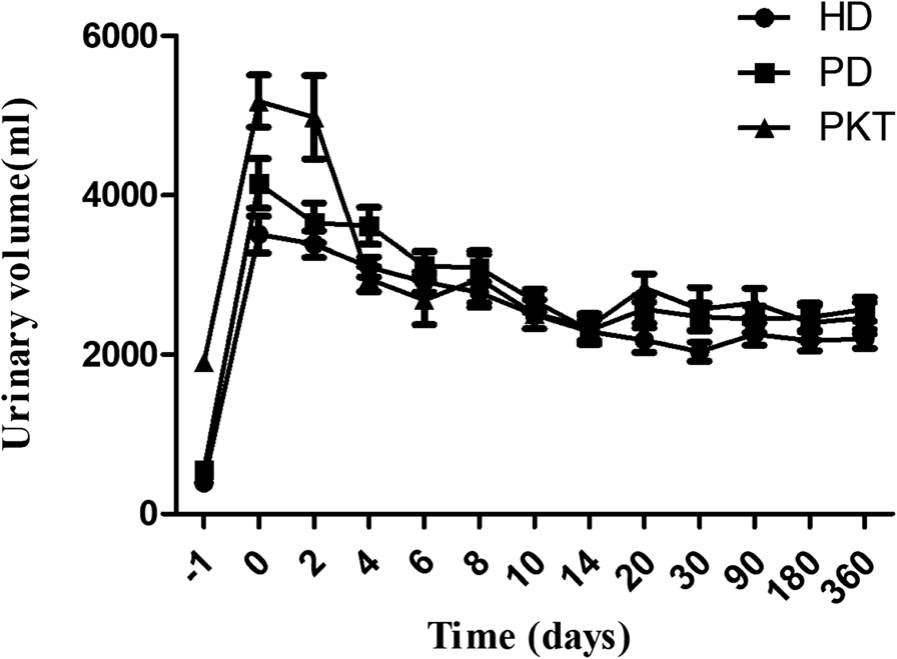
The urinary volume from pretransplant to 1 year after transplantation in PD, HD and PKT. The horizontal ordinate refers to the pretransplant time (− 1) as well as post transplantation follow-up time. During the whole follow-up period as well as pretransplant time, the urinary volume remained similar between the HD and PD patients (*p* > 0.05).PKT group was used as the control group. Data were expressed as means±S.E

The post-transplant renal function outcomes of PKT were labeled as control. In view of the HD and PD group, the post-transplant serum creatinine showed no differences throughout the follow-up between the HD and PD groups (*p* > 0.05) (Fig. 2). Pretransplant serum creatinine was higher in PD patients (*p* < 0.05). There was no differences (*p* = 0.210) in serum creatinine reduction to half of baseline in two groups, with 1.0 (1.0, 2.0) d in HD and 1.0 (1.0, 1.3) d in PD group. The serum creatinine levels at different post-transplant time points reduced statistically compared to pretransplant, which were coherent between the HD and PD groups (*p* < 0.001). There were significant differences in serum creatinine at different time points throughout the follow-up within two groups (*p* < 0.001). The 24-h urinary volume remained similar between the two groups during the follow-up period (*p* > 0.05) (Fig. 3). The mean eGFR at 1 month (68.52 ± 23.72 vs 68.04 ± 28.66, *p* = 0.902), 6 month (68.45 ± 23.15 vs 74.85 ± 22.87, *p* = 0.167) and last follow-up (69.74 ± 24.65 vs 68.54 ± 26.01, *p* = 0.737) were also similar between the two groups (Table 4).

**Table 4:** Post Kidney Transplant Laboratory Parameters of the Recipients

Characteristics	PKT group (*n* = 5)	HD group (*n* = 104)	PD group (*n* = 98)	*P*-value
Haemoglobin after transplantation at different time (g/L)				
1 month	115.6 ± 14.6	108.0 ± 20.1	103.7 ± 22.8	0.210
6 months	129.4 ± 10.1	125.3 ± 21.7	133.5 ± 20.8	0.059
The last follow-up	135.0 ± 17.8	125.2 ± 28.9	126.2 ± 26.6	0.817
Serum albumin after transplantation at different time (g/L)				
1 month	46.5 ± 2.0	42.3 ± 4.4	41.6 ± 5.3	0.394
6 months	46.9 ± 2.0	44.9 ± 3.4	45.3 ± 4.0	0.537
The last follow-up	46.1 ± 3.3	43.6 ± 5.2	43.6 ± 5.6	0.951
Cholesterol after transplantation at different time (mmol/L)				
1 month	4.9 ± 1.1	4.9 ± 1.4	4.8 ± 1.1	0.477
6 months	4.4 ± 0.5	4.8 ± 0.9	5.2 ± 0.9	0.064
The last follow-up	4.0 ± 0.8	4.8 ± 1.1	4.9 ± 1.0	0.655
Triglyceride after transplantation at different time (mmol/L)				
1 month	2.3(1.1,4.4)	1.8(1.5,2.6)	2.0(1.5,2.8)	0.533
6 months	1.5(1.1,3.0)	1.6(1.2,2.1)	2.1(1.8,2.5)	0.010*
The last follow-up	1.1(1.0,2.8)	1.6(1.1,2.3)	1.9(1.2,2.5)	0.106
Serum calcium after transplantation at different time (mmol/L)				
1 month	2.4(2.3,2.5)	2.3(2.2,2.4)	2.3(2.3,2.4)	0.374
6 months	2.4(2.2,2.7)	2.5(2.3,2.6)	2.4(2.4,2.7)	0.950
The last follow-up	2.4(2.2,2.5)	2.5(2.3,2.5)	2.4(2.3,2.6)	0.899
Serum phosphate after transplantation at different time (mmol/L)				
1 month	0.7(0.4,0.9)	0.6(0.5,0.8)	0.7(0.5,0.9)	0.291
6 months	0.9(0.9,1.2)	1.0(0.8,1.1)	0.9(0.8,1.1)	0.138
The last follow-up	0.9(0.9,1.1)	1.0(0.8,1.1)	0.9(0.8,1.1)	0.079
eGFR after transplantation at different time (mL/min/1.73m^2^)				
1 month	63.1 ± 13.8	68.5 ± 23.7	68.0 ± 28.7	0.902
6 month	58.0 ± 8.7	68.5 ± 23.2	74.9 ± 22.9	0.167
The last follow-up	57.0 ± 10.3	69.7 ± 24.7	68.5 ± 26.0	0.737

*P*-value, between hemodialysis and peritoneal dialysis group; *, statistically significant

*PKT* pre-emptive kidney transplantation, *HD* hemodialysis, *PD* peritoneal dialysis

### Other laboratory parameters post transplantation

Triglyceride levels at 6 months post transplantation were significantly higher in the PD group [PD: 2.1(1.8,2.5) vs HD:1.6 (1.2,2.1), *p* = 0.010]. At other time points the triglyceride levels were similar in two groups. There were no statistical differences between PD and HD patients in haemoglobin, serum albumin, cholesterol, triglyceride, serum calcium and serum phosphate levels throughout the follow-up period (Table 4).

### Post-transplant complications

There were only 2 infective complications in PKT group. For HD and PD group, the hyperacute rejection didn’t appear in both groups. The acute rejection rate in HD group [6.7% (7/104)] were similar to PD group [6.1% (6/98)] (*p* = 0.860). There were total 35 patients (17.3%) with DGF, 19 patients (18.3%) in HD and 16 (16.3%) in PD group. Statistically no significant differences (*p* = 0.715). Surgical and infective complications throughout the hospitalization and follow-up period were did not differ between two groups (Table 5).

**Table 5:** Post Kidney Transplant Complications

Complications	PKT group (*n* = 5)	HD group (*n* = 104)	PD group (*n* = 98)	*P*-value
Delayed recovery of graft function, n (%)	0(0)	19(18.3)	16(16.3)	0.715
Acute rejection, n (%)	0(0)	7(6.7)	6(6.1)	0.860
Surgical complications, n (%)				
Urinaryfistula	0(0)	1(1.0)	1(1.0)	1.000
Hydronephrosis	0(0)	6(5.8)	4(4.1)	0.820
Hematoma	0(0)	4(3.8)	4(4.1)	1.000
Infection, n (%)				
Cytomegalovirus	0(0)	21(20.2)	20(20.4)	0.970
JC virus	0(0)	13(12.5)	13(13.3)	0.871
BK virus	0(0)	17(16.3)	15(15.3)	0.840
Varicella zoster virus	0(0)	0(0)	1(1.0)	0.485
Fungal infectio	0(0)	4(3.8)	5(5.1)	0.927
Tuberculosis	0(0)	2(1.9)	0(0)	0.498
Urinary tract infection	1(20.0)	7(6.7)	11(11.2)	0.263
Acute bacterial pneumonia	1(20.0)	17(16.3)	12(12.2)	0.406

*P*-value, between hemodialysis and peritoneal dialysis group

*PKT* pre-emptive kidney transplantation, *HD* hemodialysis, *PD* peritoneal dialysis

### Patient mortality and graft failure

There were no death and graft failure in PKT group. There were total 7 deaths, 4 in the HD and 3 in the PD group. The patient survival rate between two groups showed no significant differences (*p* = 1.000). There were 13 graft failure, 7 in HD and 6 in PD group, and causes of graft failure were statistically different between two groups (*p* < 0.001). The graft survival rates were similar between the two groups (*p* = 0.860). The death-censored graft failure i.e. graft failure without death (3 in each of HD and PD group) was not different between the two groups (*p* = 1.000) (Table 6).

**Table 6:** The Patient and Graft Survival Rates throughout Follow-up Time and the Causes of Graft Failure

Characteristics	PKT group (*n* = 5)	HD group (*n* = 104)	PD group (*n* = 98)	*p*-value
Transplatation outcomes, %(n)				
Patient survival	100.0(5/5)	96.2(100/104)	96.9(95/98)	1.000
Graft survival	100.0(5/5)	93.3(97/104)	93.9(92/98)	0.860
Death-censored graft survival	100.0(5/5)	97.1(101/104)	96.9(95/98)	1.000
Causes of graft failure, % (n)				< 0.001*
Acute rejection	0(0/0)	0(0/7)	28.6(2/7)	
Severe infection	0(0/0)	28.6(2/7)	57.1(4/7)	
Primary failure	0(0/0)	0(0/7)	14.3(1/7)	
Surgical complications	0(0/0)	57.1(4/7)	0(0/7)	
Others	0(0/0)	14.3(1/7)	0(0/7)	

*P*-value, between hemodialysis and peritoneal dialysis group; *, statistically significant

*PKT* pre-emptive kidney transplantation, *HD* hemodialysis, *PD* peritoneal dialysis

The cox proportional hazards model showed pretransplant dialysis modality (HD and PD) had no correlation with patient survival or graft failure or death-censored graft survival After adjusting for other related multiple risk factors, the PD patients had similar rates of graft failure compared with HD in multivariate cox proportional hazards analysis (Table 7). When separately analyzed for HD and PD groups, the surgical complications in HD patients were independent stimulating factors of graft failure and DGF was an independent factor inversely correlated with graft survival in PD patients (Table 8).

**Table 7:**
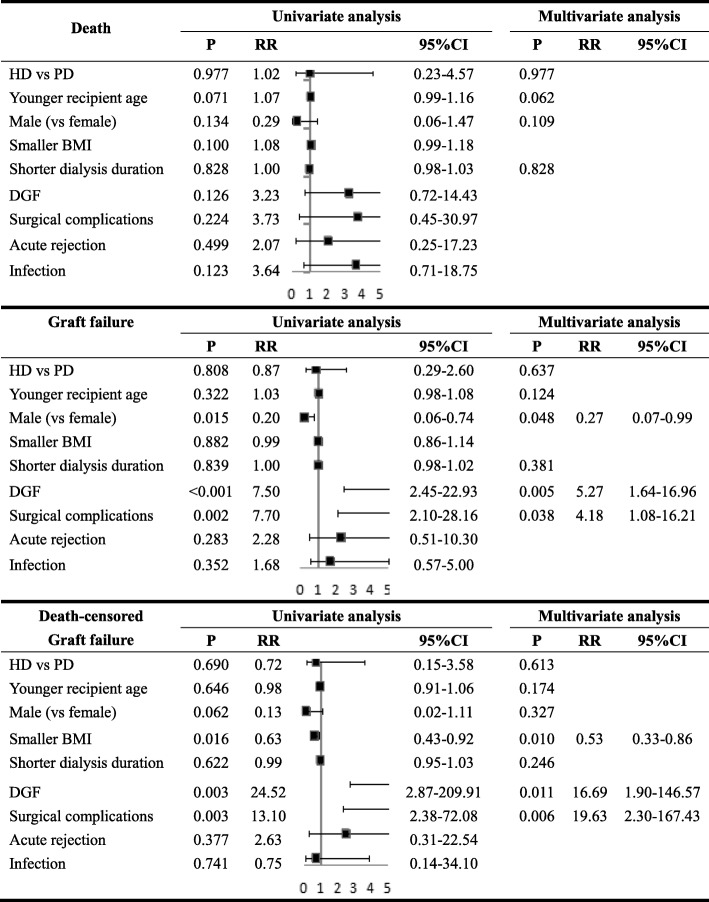
The Univariate and Multivariate Analysis for Effects of HD vs PD and Other Factors on Outcomes of Non-preemptive Kidney Transplantation

*HD* hemodialysis, *PD* peritoneal dialysis, *P p* value, *RR* relative risk, *CI* confidence interval, *BMI* body mass index, *DGF* delayed graft failure

**Table 8:** Univariate and Multivariate Analysis for Effects of Factors on Graft Failure of Kidney for Non-preemptive Transplantation According to Dialysis Modality

	Univariate analysis			Multivariate analysis		
	P	RR	95% CI	P	RR	95% CI
Graft failure in HD						
Younger recipient age	0.731	0.99	0.92–1.06	0.947		
Male (vs female)	0.100	0.25	0.05–1.30	0.290		
Smaller BMI	0.761	1.02	0.89–1.17			
Shorter dialysis duration	0.211	0.96	0.91–1.02	0.250		
DGF	0.017	6.23	1.40–27.85	0.588		
Surgical complications	< 0.001	26.12	5.70–119.80	< 0.001	26.12	5.70–119.80
Acute rejection	0.629	0.044	–			
Infection	0.472	0.55	0.11–2.83			
Graft failure in PD						
Younger recipient age	0.071	1.06	0.99–1.15	0.105		
Male (vs female)	0.084	0.15	0.02–1.29	0.112		
Smaller BMI	0.420	0.88	0.64–1.20			
Shorter dialysis duration	0.373	1.01	0.99–1.04	0.962		
DGF	0.010	9.39	1.72–51.29	0.010	9.39	1.72–51.29
Surgical complications	0.700	0.05	–	0.708		
Acute rejection	0.036	6.15	1.12–33.67			
Infection	0.058	7.96	0.93–68.11			

*HD* hemodialysis, *PD* peritoneal dialysis, *P p* value, *RR* relative risk, *CI* confidence interval, *BMI* body mass index, *DG* delayed graft failure

## Discussion

Up to 30–40% of patients are can be effectively treated by PD, far away from current 11% [16] and many suitable PD candidates are treated with HD [17]. The use of PD is lower than HD owing to the aging of dialysis population, comorbidity and social conditions that make home PD difficult. More studies are needed to clarify the identical or even better function of PD compared with HD.

Our results indicated both the immediate and long-term renal function, the serum creatinine and urine output, were similar between the HD and PD, consistent with other studies [6, 11]. In our study, the baseline serum creatinine was higher in PD than HD patients. The HD just before the transplantation could have lowered the serum creatinine. In contrast, PD lowers creatinine in moderate ways and high baseline serum creatinine in PD patients doesn’t mean it is inferior to HD in creatinine reduction. This could be the reason why both groups had similar renal function after transplantation.

We had no significant differences in the incidence of AR, which is in line with recent studies [3, 6, 11, 12]. This may be due to the availability of and rational novel immunosuppressive protocols nowadays. Our study shows similar incidence of DGF in both PD and HD patients, as reported by others [6, 10]. We also found that DGF was inversely associated with the graft survival and death-censored graft survival regardless of dialysis modality. The DGF is associated with greater risk of patient death in addition to graft and death-censored graft failure [18]. Ischemic-reperfusion of donated kidney caused by postischemic acute tubular necrosis and interstitial inflammation results in DGF [19]. The PD patients has lower incidence of DGF in comparison with the HD [9, 11, 12]. This could be due to better hydration status and preservation of residual renal function (RRF) in PD patients [13]. Additionally, the PD patients has less oxidative stress which can exacerbate ischemic-reperfusion injury in kidney compared with HD patients [20].

Our results shows similar patient, graft and death-censored graft survival rate in PD and HD groups, consistent with most other studies [5, 6, 12]. Earlier, in a large cohort study of 22,776 patients concluded a higher rate of early graft failure (during the first 3 months after KT) in PD, possibly due to higher incidence of early graft thrombosis [18]. While the long-term graft failure and patient mortality remained similar. Some studies report PD had better patient survival, better quality of life, better nutritional status and fewer blood transfusions [9–11]. The differences might be associated with the different sample size and the follow-up time.

Some of the limitations of our study could be a single-center and inclusion of first transplantation from DCD only may not be applicable to all renal transplantations. In addition, the study variables of donors were incomplete, with some statistically analysis based on the less data compared to recipients. Besides, the pre-emptive kidney transplantation group in the cohort had only 5 patients, with people too less to be statistically comparative analyzed, finally simply summarized and displayed as the control group. And the follow-up period was not long enough, with further study and investigations to go on.

## Conclusions

The choice of dialysis modality, HD or PD, prior to kidney transplantation had no influences on the patient, graft and death-censored graft survival. The immediate and long-term complications after transplantation, and renal function between the two groups were similar. Thus we can conclude that PD is equally good with potential for wider applicability as pretransplant modality of dialysis.

## Abbreviations

ALT: Alanine aminotransferase
AR: Acute rejection
BMI: Body mass index
CyA: Cyclosporine A
DCD: Donor after cardiac death
DGF: Delayed graft function
eGFR: Estimated glomerular filtration rate
ESRD: End-stage renal disease
FK: Tacrolimus
HBV: Hepatitis B virus
HD: Hemodialysis
HDL: High-density lipoprotein
ICU: Intensive care unit
KT: Kidney transplantation
LDL: Low-density lipoprotein
MMF: Mofetil mycofenolate
P: *p* value
PD: Peritoneal dialysis
PKT: Pre-emptive kidney transplantation
PTH: Parathyroid hormone
RR: Relative risk
SD: Standard deviations
St: Steroids
